# Integrating frailty into decision-making for disease-modifying therapies in Alzheimer’s disease: a proposed expert-opinion based approach

**DOI:** 10.1016/j.tjpad.2026.100666

**Published:** 2026-09-04

**Authors:** Giuseppe Bellelli, Ovidio Brignoli, Marco Canevelli, Antonio Cherubini, Andrew Clegg, Bruno Dubois, Maria Cristina Ferrara, Laura Fratiglioni, Giovanni B. Frisoni, Giulia Grande, Frank Jessen, Sean P Kennelly, Alberto Magni, Martina Marelli, Alessandra Marengoni, Nicolás Martínez Velilla, Susan D. Shenkin, Marc Suárez-Calvet, Suzanne Timmons, Alessandro Padovani

**Affiliations:** a University of Milano Bicocca and Acute Geriatric Unit, IRCCS San Gerardo dei Tintori, 20900 Monza, Italy; b General Practitioner, Italian College of General Practitioners, 50144 Firenze, Italy; c Department of Human Neuroscience, Sapienza University, Rome, Italy and Aging Research Center, Department of Neurobiology, Care Sciences and Society, Karolinska Institutet and Stockholm University, 11330 Stockholm, Sweden; d Geriatria, Accettazione geriatrica e Centro di ricerca per l’invecchiamento. IRCCS INRCA, 60127 Ancona, Italy; e Dipartimento di Scienze Cliniche e Molecolari, Università Politecnica delle Marche, 60126 Ancona, Italy; f Academic Unit for Ageing and Stroke Research, Bradford Institute for Health Research Bradford Institute for Health Research, BD96RJ Bradford, UK; g Institut de la Mémoire et de la Maladie d'Alzheimer, IM2A, Groupe Hospitalier Pitié-Salpêtrière, Sorbonne Université, 75013 Paris, France; Institut du Cerveau et de la Moelle Épinière, UMR-S975, INSERM, Hôpital de la Pitié-Salpêtrière, 75013 Paris, France; h Aging Research Center, Department of Neurobiology, Care Sciences and Society, Karolinska Institutet and Stockholm University, 11330 Stockholm, Sweden; i Centre de la mémoire, Department of Rehabilitation and Geriatrics, Geneva University and University Hospitals, 1211 Geneva, Switzerland; j Klinik und Poliklinik für Psychiatrie und Psychotherapie, Uniklinik Köln, Medizinische Fakultät, Kerpener Straße 62, 50937, Köln, Deutschland, Germany; k Institute of Memory and Cognition, Tallaght University, D24NR0A Dublin, Ireland; Dept of Medical Gerontology, School of Medicine, Trinity College Dublin, D02PN40 Dublin, Ireland; l Department of Clinical and Experimental Sciences, University of Brescia, 25121 Brescia, Italy; m Navarre Health Service (SNS-O), Navarre University Hospital (HUN), Department of Geriatrics, Navarrabiomed, Navarre Public University (UPNA), Navarra Institute for Health Research (IdiSNA), 31008 Pamplona, Spain and CIBER of Frailty and Healthy Aging (CIBERFES), Instituto de Salud Carlos III, 28029 Madrid, Spain; n Ageing and Health, and Advanced Care Research Centre, Usher Institute, University of Edinburgh, EH164UX Edinburgh, Scotland, UK; o Barcelona Brain Health Center, Barcelonaβeta Brain Research Center (BBRC), Pasqual Maragall Foundation, 08005 Barcelona, Spain and Barcelona Brain Health Center, Department of Neurology, Hospital del Mar, 08003 Barcelona, Spain; p Centre for Gerontology and Rehabilitation, School of Medicine, University College Cork, T12XH60 Cork, Ireland; q Department of Clinical and Experimental Sciences, University of Brescia, and Neurobiorepository and Laboratory of Advanced Biological Markers, University of Brescia and ASST Spedali Civili Hospital, 25123 Brescia, Italy

**Keywords:** Frailty, Alzheimer’s disease, Older adults, Disease-modifying therapy

## Abstract

The introduction of disease-modifying therapies for Alzheimer’s disease (AD-DMTs) is reshaping clinical practice, raising critical questions about patient selection, diagnostic pathways, treatment appropriateness, and equity of access. Frailty, a multidimensional condition of reduced physiological reserve and increased vulnerability to stressors, is common in older adults with AD, yet has not been systematically assessed in AD-DMTs trials, limiting the generalizability of trial findings to real-world populations.

In this review, we examine the role of frailty in the emerging era of AD-DMTs, summarizing evidence on its prevalence and prognostic relevance, approaches to its assessment, and its potential impact on treatment safety and effectiveness. We propose that regular frailty assessment should inform decision-making in both clinical trials and clinical practice, while frailty-informed management—including medication review and multidomain interventions—may support more appropriate, individualized care.

## 1 Introduction

The advent of disease-modifying therapies for Alzheimer’s disease (AD-DMTs) is reshaping clinical practice, raising new questions about patient selection, treatment appropriateness, and equity of access. In this evolving landscape, frailty is a key yet inconsistently addressed factor in therapeutic decision-making, particularly among older adults, who make up the largest group of patients with Alzheimer’s dementia [1,2].

This article, developed by a multidisciplinary panel of European geriatricians, neurologists, psychiatrists, and primary care physicians, aims to provide a clinically grounded conceptual framework for frailty in the emerging era of AD-DMTs. We first examine frailty as a unifying measure of biological aging. We then discuss its clinical relevance, areas of uncertainty, ethical considerations in the context of AD-DMTs, and its potential as a therapeutic target to improve health outcomes in Alzheimer’s disease (AD). Finally, we discuss how comprehensive assessment, including frailty, may inform clinical decision-making.

We propose moving from a traditional brain-centred paradigm—where diagnostic and treatment indications rely mainly on disease-centred neuropsychiatric criteria—to a new person-centred approach in which frailty assessment holistically informs the risk–benefit balance of AD-DMTs and supports multidomain interventions targeting not only AD but also frailty, associated geriatric syndromes, and coexisting long-term conditions. The article concludes by outlining future directions for research, trial design, and healthcare system readiness.

## 2 Current knowledge on frailty and Alzheimer’s disease

### 2.1 Frailty: a unifying measure of biological aging

Population ageing is accelerating worldwide, with the fastest growth occurring among the oldest-old. This demographic transition is accompanied by substantial heterogeneity in ageing trajectories, as individuals of the same chronological age may differ markedly in health status [3]. In this context, chronological age alone is insufficient to guide clinical decision-making, particularly in an era oriented toward precision medicine [4]. Frailty has emerged as a clinically meaningful construct that captures this heterogeneity. It is commonly defined as a multidimensional condition characterized by reduced physiological reserve and increased vulnerability to stressors, leading to a higher risk of adverse health outcomes [5]. Although frailty is not exclusively related to aging, its prevalence increases markedly with advancing age, affecting approximately one in four individuals aged ≥80 years and nearly half of those aged ≥90 years worldwide [6]. Importantly, it is a dynamic condition. In a population-based cohort of 1,339 older adults (mean age 72.7 years; 9-year follow-up), multistate models identified 1,931 transitions between frailty states. Transitions toward less severe states were common, with remission/improvement reaching 20% at 3 years, indicating substantial reversibility of frailty [7]. These findings challenge the view of frailty as an inevitably progressive condition and highlight opportunities for intervention.

### 2.2 Operationalizing frailty

Two major conceptual models have been proposed to operationalize frailty.

The first conceptualizes frailty as a clinical syndrome, operationalized through Fried’s Frailty Phenotype (FFP) [8]. This model reflects impaired metabolic regulation and altered physiological response to stress and is defined by five core features: exhaustion, weakness, slowness, low physical activity, and unintentional weight loss. Individuals are classified as robust when none of these features are present, prefrail when one or two criteria are met, and frail when three or more criteria are present. Importantly, the derivation of the frailty phenotype excluded individuals with Mini Mental State Examination scores below 18/30, which should be considered when applying this model in populations with significant cognitive impairment [8].

The second model conceptualizes frailty as the cumulative accumulation of health deficits across multiple domains, including diseases, symptoms and signs, functional and cognitive impairments, and laboratory abnormalities [9]. Within this framework, frailty is operationalized through a Frailty Index (FI), calculated as the ratio between the number of deficits present in an individual and the total number of deficits considered [5]. A minimum of 25-30 health deficits is generally recommended to ensure construct validity, although the original FI incorporated approximately 70 items [9]. In most applications, frailty is defined when 20% of the considered deficits are present [5].

Following these two models, a wide range of instruments has been developed and validated to assess frailty in clinical and research settings, ranging from brief screening tools to multidimensional assessments [10,11]. For the present review, we conducted a PubMed search from database inception to Dec 30, 2025, using the terms (“frail*” AND “Alzheimer”) without language restrictions. Of the 704 records identified, 50 articles were included after independent screening by two reviewers. Frailty was assessed using the FFP model in 15 studies [[12], [13], [14], [15], [16], [17], [18], [19], [20], [21], [22], [23], [24], [25], [26]] and through deficit accumulation models in 20 studies [[27], [28], [29], [30], [31], [32], [33], [34], [35], [36], [37], [38], [39], [40], [41], [42], [43], [44], [45]]. Three additional studies used both approaches [[46], [47], [48]], while only one applied a different tool [49].

Although conceptually and operationally distinct, both the FFP and deficit accumulation models likely capture a shared underlying biological construct. Frailty reflects the cumulative dysregulation of multiple physiological systems, which may be driven by processes such as chronic low-grade inflammation (“inflammaging”), cellular senescence, mitochondrial dysfunction, and endocrine dysregulation [5]. These processes contribute to multisystem decline and impair the adaptive mechanisms that maintain physiological homeostasis in response to internal or external stressors [50]. In other words, frailty can be interpreted as a global marker of reduced systemic resilience.

Importantly, frailty should be conceptualized as a continuum rather than as a dichotomous state. It reflects graded reductions in physiological reserve that cannot be adequately captured by a single threshold, and dichotomous classifications can therefore oversimplify the clinical complexity of older adults.

### 2.3 Frailty instruments for individuals with AD

The relationship between frailty and cognitive decline is likely complex and bidirectional, as cognitive decline may both result from and signal underlying frailty. There is consistent evidence that frailty—across both phenotype- and deficit accumulation–based approaches—is associated with an increased risk of incident dementia [24,51]. However, it remains unclear whether specific frailty tools can predict cognitive decline, disease progression independent of AD pathology, or response to AD-DMTs. A potential first step would be to systematically combine cognitive and frailty assessment, as considering cognitive impairment alongside physical frailty may improve its predictive validity [52]. As previously suggested, the progression of cognitive decline should also be considered as a potential marker of increased vulnerability, when interpreted alongside other frailty domains [53]. However, the complementary value of assessing cognition and frailty does not necessarily support the adoption of “cognitive frailty” as a distinct construct. Indeed, the conceptual and clinical added value of such a construct remains debated, particularly given the inherently multidimensional nature of frailty [54,55]. Moreover, the applicability of “cognitive frailty” concept to AD-DMT context may be limited, as its traditional definition relies specifically on the FFP, without clear evidence that this should be preferred over other frailty tools, and on MCI, thereby excluding individuals with dementia [56].

From a public health and service planning perspective, frailty instruments should support population-level risk stratification and resource allocation across care pathways, providing a transversal measure of vulnerability beyond individual diseases. In this context, FI-based approaches are particularly suitable, as they leverage routinely collected data and can be easily implemented at scale in primary care. In the United Kingdom, for example, the electronic Frailty Index (eFI) and the Clinical Frailty Scale (CFS) are widely used by general practitioners [57,58], while in Italy, the Primary Care Frailty Index (PC-FI) [59] has been incorporated into many primary care electronic medical record systems.

By contrast, phenotype-based models such as the FFP are better suited to functional, patient-level assessment, particularly when detailed evaluation of physical performance is feasible. These models may be preferred when a clearer distinction between frailty, disability, and comorbidity is required.

In research contexts, the use of multiple frailty instruments based on different conceptual models may be particularly informative, as this allows comparison of predictive performance and improves characterization of vulnerability across heterogeneous populations.

Other validated instruments, including the Tilburg Frailty Indicator, Groningen Frailty Indicator, and PRISMA-7, may also be considered for clinical practice and research, depending on the specific objectives and clinical setting [60,61].

Table 1 outlines the biological basis, prevalence, and metrics of frailty along with their clinical implications in the context of cognitive impairment.

**Table 1:** Scientific framework and assessment of frailty in Alzheimer’s disease.

	Description	Clinical implication
**Biological basis**	Characterized by “inflammaging” (chronic low-grade inflammation), mitochondrial dysfunction, cellular senescence, and hormonal dysregulation.	Frailty is a marker of biological ageing which is often poorly captured by chronological age.
**Prevalence in AD**	Affects approximately 32% of community-dwelling AD patients.	Frailty is the rule rather than the exception in real-world clinical practice of older people with cognitive impairment.
**Fried’s Frailty Phenotype (FFP)**	Based on 5 physical criteria: exhaustion, weakness (handgrip strength), slowness (gait speed), low activity, and weight loss. Scoring: 0 = robust; 1–2 = prefrail; ≥3 = frail.	Useful for identifying physical vulnerability and risk of rapid decline.
**Rockwood** **Frailty Index (FI)**	A ratio of accumulated health deficits (diseases, symptoms, laboratory abnormalities, and functional impairments). Scoring**:** continuous from 0 to 1, with higher values indicating greater frailty	Better at capturing the multisystemic complexity of older patients.
**Clinical Frailty Scale**	A 9-level scale based on comorbidity, functional status, and level of dependence, ranging from 1 (very fit) to 9 (terminally ill)	A practical bedside instrument for rapid stratification of overall vulnerability and for informing clinical decision-making in older adults.
**Longitudinal course**	Evidence shows that frailty is a dynamic and modifiable condition; it can be reversed or improved through targeted multidomain interventions.	Provides an opportunity for combining pharmacological and non-pharmacological treatments.

## 3 Frailty at the crossroads of AD-DMT decision-making

Although a formal discussion has yet to be articulated in the scientific literature and among expert communities, frailty lies at an important intersection with decision-making around AD-DMT, including patient selection, risk stratification and treatment monitoring. Current eligibility criteria for AD-DMTs are largely based on biomarker and clinical definitions of AD, with limited consideration of broader vulnerability profiles such as frailty. Persons living with frailty (PLwF) may be perceived as suboptimal candidates for DMTs, a view often driven by concerns regarding safety, treatment tolerability, and potential futility in biologically vulnerable populations.

At the same time, the limited evidence currently available leaves several questions unresolved, warranting further investigation and offering opportunities for future research and clinical guidance.

### 3.1 Clinical concerns

One of the main concerns relates to the potential susceptibility of PLwF to iatrogenic harm. Frailty has consistently been associated with a higher risk of adverse drug reactions (ADRs) and medical complications across multiple therapeutic contexts. For example, in hospitalized older adults, patients classified as frail using a 34-item FI were found to have approximately twice the risk of ADR compared with non-frail individuals [62]. Anti-amyloid therapies are known to increase the risk of amyloid-related imaging abnormalities (ARIA), both haemorrhagic (ARIA-H) and oedematous (ARIA-E) [63]. Although symptomatic ARIA are relatively uncommon, reduced physiological reserve in PLwF may increase vulnerability to treatment-related complications, particularly in the presence of high cerebral amyloid burden, which itself increases the risk of ARIA [[64], [65], [66], [67]]. A lower amyloid burden for a given degree of clinical impairment could theoretically reduce the risk of ARIA, although the pro-inflammatory milieu associated with frailty might conversely increase susceptibility during amyloid clearance. Notably, in both the lecanemab and donanemab trials, participants achieving earlier amyloid clearance tended to be older, suggesting a complex interplay between chronological age, biological vulnerability, and treatment response [65,66].

However, this hypothesis remains largely untested, as studies specifically examining the interaction between frailty and ARIA are currently lacking. Frailty was not systematically assessed in registrational trials [[65], [66], [67]], and only a small fraction of real-world patients were enrolled, mostly being younger, healthier, early AD patients [68].

Frailty is also a well-established risk factor for delirium [69], which is itself associated with accelerated cognitive decline, independently of underlying AD pathology [[70], [71], [72]]. Delirium – often clinically described as “confusional state”– has been reported in patients developing symptomatic ARIA during treatment with DMTs [66]. However, the temporal and biological relationships between frailty, delirium, and ARIA remain unclear. Although relatively uncommon, delirium may represent both a direct manifestation of ARIA in this context, an indicator of underlying frailty-related vulnerability, or the result of their interaction. This raises the possibility that frailty may contribute to increased clinical complexity in patients receiving AD-DMTs.

Finally, mixed neuropathology is common in older adults and may be particularly relevant in PLwF, in whom cognitive impairment may reflect both AD and non-AD mechanisms [29,73,74]. This should be carefully considered when assessing potential eligibility for AD-DMTs.

### 3.2 Areas of uncertainties and “grey zones”

Whether and how frailty can interact with brain structure remains incompletely understood. Longitudinal studies indicate that frailty predicts the development of AD dementia independently of established AD pathophysiological hallmarks [36,70]. Frailty has also been associated with structural brain changes, including increased white matter hyperintensities [75], and reduced brain volume [76]. Autopsy studies further suggest that PLwF may exhibit decreased resistance and resilience to neuropathological changes, resulting in heightened cognitive impairment even in the presence of low levels of AD pathology [77]. Consistent with these findings, frailty was found to amplify the cognitive impact of amyloid/tau/neurodegeneration (AT[N]) pathology in a large Alzheimer’s Disease Neuroimaging Initiative cohort (n=1,711 participants), independently of demographics and APOE ε4 status. However, limitations in the construction of the FI used in this study should be acknowledged [33]. Together, these findings suggest that even modest levels of AD–related pathology may be sufficient to precipitate cognitive decline in PLwF [29,77,78].

Emerging evidence also supports a continuous crosstalk between skeletal muscle and brain. Sarcopenia—defined as the age-related loss of muscle mass and function and a key component of the FFP [8]—has strongly been linked to brain health. In a large UK Biobank study including more than 33,000 individuals, sarcopenic traits were associated with lower cognitive performance and widespread brain structural differences (including reduced cortical thickness, lower white-matter integrity, and smaller brain volumes, particularly within sensorimotor regions), independently of major confounding factors such as socioeconomic status, education, lifestyle, and physical activity. Brain structural alterations partly mediated the association between sarcopenia and cognition [79]. Consistent with this muscle–brain axis, circulating biomarkers of AD pathology—such as plasma p-tau181, p-tau217, and neurofilament light chain (NfL)—have been associated with faster long-term decline in handgrip strength over 15 years, independent of, or alongside, cognitive decline [80].

Overall, these observations suggest that frailty may contribute to cognitive decline through interacting biological mechanisms which are only partially dependent on amyloid-β accumulation [81]. This perspective aligns with emerging reinterpretations of the amyloid cascade hypothesis, which increasingly consider amyloid-β as one component of a broader network of interacting pathological processes rather than as a sole driver of the disease [82].

These observations have implications for AD-DMTs use. Even partial amyloid-β reduction may yield clinically meaningful cognitive benefit in individuals with high amyloid-driven vulnerability. By contrast, in frailty—where decline may reflect multisystem and non-amyloid mechanisms—treatment effects may be limited.

These potential benefits must be weighed against treatment burden, including repeated infusions, serial MRI monitoring, travel, financial costs, and caregiver involvement, as well as the complexity of managing intercurrent clinical events and concomitant treatments (such as antithrombotic therapy) in PLwF [68,83]. Emerging real-world experience with AD-DMTs underscores the importance of careful patient selection and structured multidisciplinary care accounting for comorbidities, concomitant medications, treatment adherence, and caregiver support [84,85]. Although not yet systematically evaluated in this context, frailty could provide an integrated measure of vulnerability that complements established treatment-specific criteria, helping to contextualize the overall benefit–burden balance and support person-centred treatment decisions. Incorporating frailty into health-economic evaluations may also better capture QALYs, healthcare costs, and broader societal consequences, including caregiver burden [86].

### 3.3 Ethical considerations

As frailty is common in older adults, a growing proportion of individuals seeking access to AD-DMTs are likely to be frail or pre-frail. However, this has not been systematically evaluated in clinical trials, making it unclear whether PLwF were under-represented, adequately represented, or inadvertently included without recognition, thereby limiting interpretation of treatment safety and effectiveness and raising concerns about potential inequities in evidence generation.

In this context of limited evidence, clinical decision-making becomes particularly challenging. Excluding individuals on the basis of frailty risks restricting access for a substantial and clinically relevant segment of the target population [87]. Moreover, it risks conflating biological vulnerability with therapeutic futility [88,89]. Conversely, failure to recognize frailty may expose patients to treatment without adequate consideration of their resilience and risks. If such treatments ultimately prove ineffective in this population, additional ethical concerns may also arise regarding the appropriate use of limited healthcare resources.

The European Medicines Agency has recommended consideration of frailty assessment in clinical trials involving older adults [[90], [91], [92]], and expert consensus with a dementia focus has further emphasized the need for systematic frailty assessment and its routine evaluation in individuals living with cognitive impairment [93]. However, these recommendations lack consistent implementation and widespread adoption across AD-DMT registrational trials. This gap further limits the interpretability of available evidence and raises concerns about inequities in evidence generation [94].

In clinical practice, this evidence gap may translate into uncertainty and variability in treatment decisions. Frailty may be interpreted inconsistently—as a contraindication, a marker of increased risk requiring caution, or a dimension to be balanced against potential benefit—without clear empirical guidance.

Thus, the central challenge is not simply whether PLwF were included in trials, but how frailty should be explicitly integrated into evidence generation and clinical decision-making. Without such integration, treatment decisions risk being driven by implicit assumptions rather than systematic assessment, potentially resulting in both inappropriate exclusion and insufficiently informed treatment of biologically vulnerable patients.

## 4 Towards a new care model for older adults: frailty assessment as a clinical compass

### 4.1 Frailty assessment as a guide for clinical decision-making

**Fig. 1:**
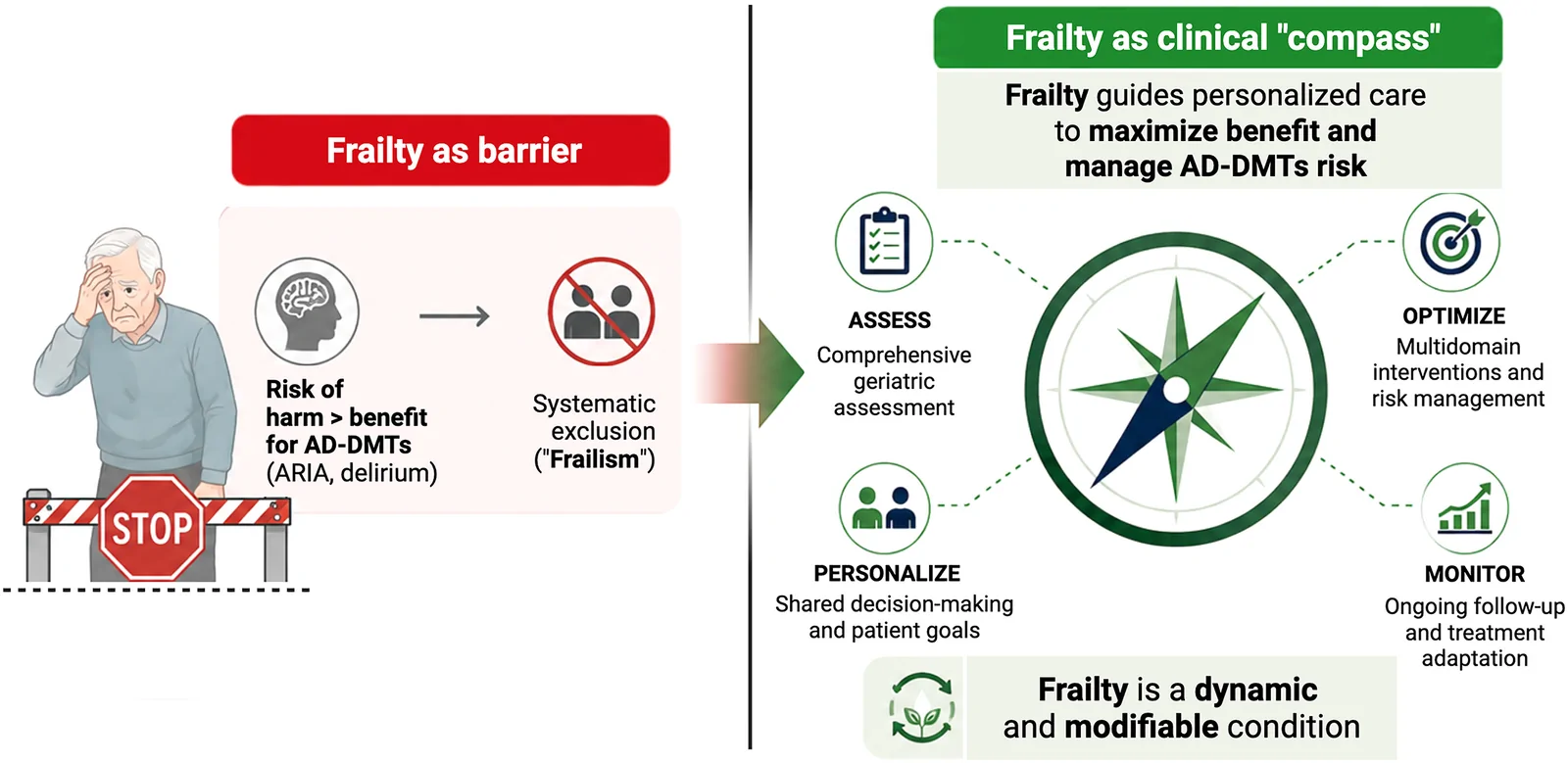
Frailty and disease-modifying therapies: from barrier to clinical “compass”.

**Legend:** The left panel illustrates a barrier-oriented view of frailty, whereby concerns about treatment-related harms, including amyloid-related imaging abnormalities (ARIA) and delirium, may lead to systematic exclusion of frail individuals from Alzheimer’s disease – disease-modifying therapies, potentially resulting in “frailism”. The right panel reframes frailty as a clinical “compass”, that, through comprehensive geriatric assessment, shared decision making, multidomain interventions, and longitudinal monitoring, support personalized and appropriate treatment strategies. (Figure created with BioRender - https://www.biorender.com).

From this expert perspective, frailty assessment should be conceptualized as a clinical compass guiding decision-making related to AD-DMTs (Fig. 1). Integrating frailty into routine evaluation supports a truly person-centred, rather than disease-centred approach. By capturing biological vulnerability, functional reserve, and resilience to stressors, frailty assessment allows clinicians to move beyond a narrow focus on cognitive scores or biomarkers and to better contextualize therapeutic decisions within the individuals’ overall health trajectory.

Systematic frailty assessment also enables longitudinal monitoring. Changes in frailty status over time may signal meaningful shifts in health which are not reflected by cognitive measures alone, offering a more sensitive marker of treatment impact, tolerability, and evolving risk–benefit balance [95]. In clinical practice, frailty assessment is typically embedded within the Comprehensive Geriatric Assessment (CGA), a standardized, evidence-based multidimensional evaluation of medical, functional, cognitive, and psychosocial domains. CGA supports the interpretation of these dynamic changes, facilitates shared decision-making, and informs tailored, multidomain interventions. It also allows early detection of subclinical deterioration or improvement across key domains, including mobility, function, nutrition, and social vulnerability, which are highly relevant to older adults and their caregivers [96].

Although evidence supporting frailty-informed care in the AD-DMT setting is currently lacking, experience from other clinical fields supports its potential value in guiding disease-specific management. CGA-driven models of care have improved the management and clinical outcomes of frail older adults across different healthcare settings, while geriatric assessment–based approaches in oncology and perioperative care have influenced treatment decisions and, in some studies, reduced treatment-related toxicity and complications [97]. Similarly, cardiovascular trials suggest that frailty assessment may refine rather than restrict therapeutic decision-making, as treatment benefits can be preserved, or even greater in absolute terms, among patients with higher degrees of frailty [98,99]. Whether integrating frailty assessment into AD-DMT pathways translates into improved patient-centred outcomes, healthcare utilization, or cost-effectiveness remains to be established and represents an important priority for future research.

### 4.2 Frailty as a target for interventions to improve cognition in AD

**Fig. 2:**
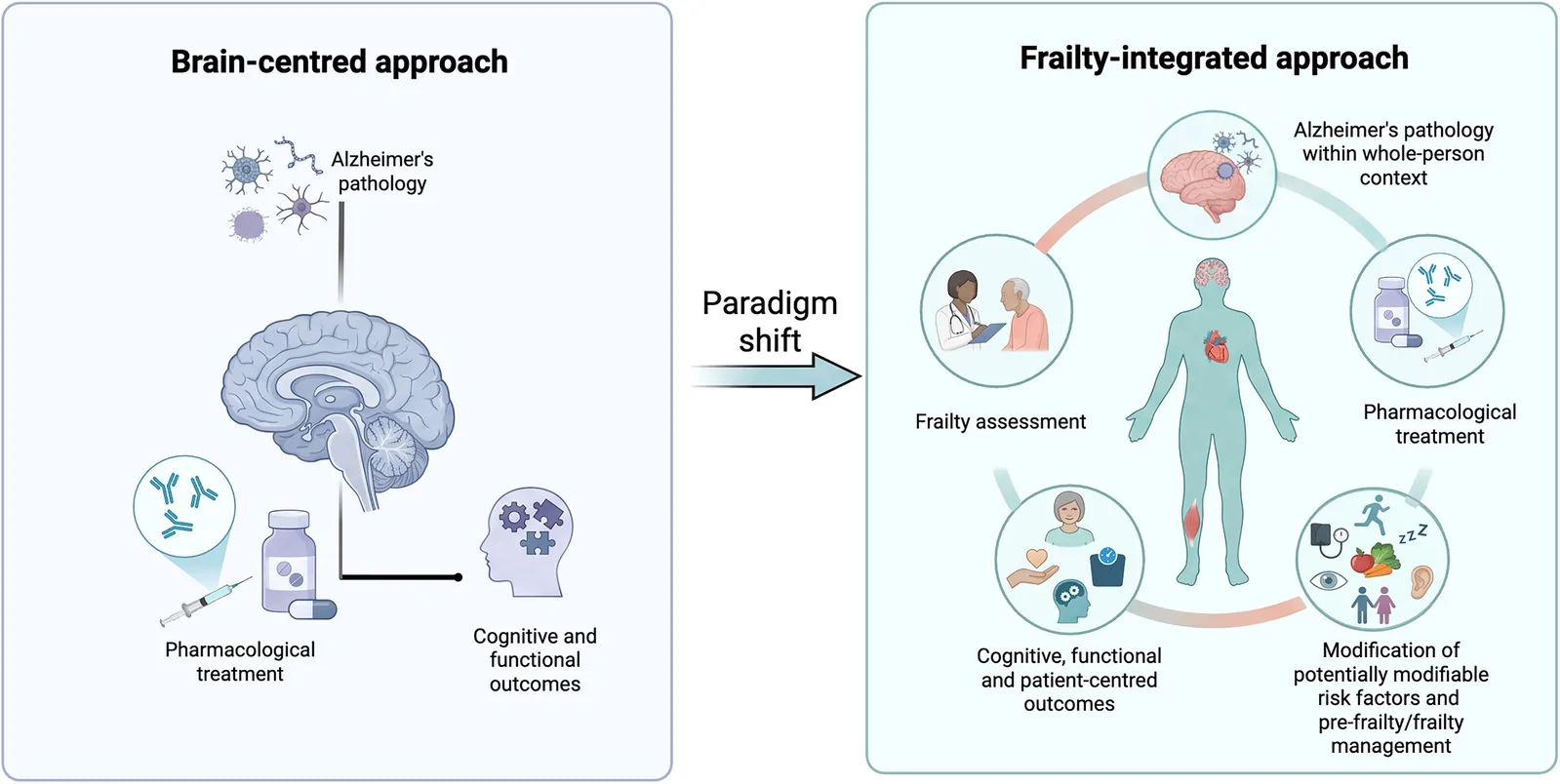
From brain-centred to whole-person care in Alzheimer’s disease.

**Legend:** The left panel illustrates a predominantly brain-centred model of Alzheimer’s disease care, focused primarily on Alzheimer’s pathology, pharmacological treatment, and cognitive and functional outcomes. In contrast, the right panel illustrates a frailty-integrated, whole-person approach, in which Alzheimer’s pathology is considered within the broader context of the individual. This approach incorporates frailty assessment, management of potentially modifiable risk factors, and pharmacological treatment, while considering cognitive, functional and patient-centred outcomes. (Figure created with BioRender - https://www.biorender.com).

Traditionally, therapeutic models for AD have focused on the pathophysiological cascade from amyloid-β deposition to tau hyperphosphorylation and neurodegeneration across the continuum from normal cognition to Mild Cognitive Impairment (MCI) and dementia [100,101]. However, growing evidence of crosstalk between systemic health and brain function suggests that complementary strategies may help improve cognition in PLwF and AD (Fig. 2) [102]. This approach may enhance the brain’s resilience to amyloid burden and other pathological processes contributing to cognitive decline.

Within this perspective, stepwise approaches prioritising low-cost interventions aimed at increasing such resilience should be considered before, or at least alongside, plaque-removal strategies, with attention to evaluating the effectiveness of each component of these interventions.

This rationale is supported by growing evidence that frailty is a potentially reversible condition. Multidomain interventions targeting physical activity, nutrition, vascular risk factors, mental stimulation, and social engagement have shown beneficial effects on cognitive outcomes in cognitively unimpaired but at-risk older adults [103]. Beneficial effects have also been observed in individuals with MCI after a 12-week Vivifrail exercise programme [104]. Within this framework, frailty should be regarded not only as a risk marker but also as a potential therapeutic target. CGA may help identify reversible contributors and guide personalized interventions aimed at improving overall resilience [105,106]. Indeed, multidomain interventions targeting modifiable lifestyle and vascular risk factors have shown beneficial effects on cognitive outcomes in older adults at increased risk of cognitive decline [103,107]. Embedding these interventions within care pathways alongside AD-DMTs may therefore help maximise overall therapeutic benefit while mitigating risks associated with biological vulnerability. However, dedicated studies are needed to assess their impact across multiple geriatric domains—including cognition, mobility, falls, and continence–as well on the effectiveness and safety of AD-DMTs in PLwF.

### 4.3 Interdisciplinary collaboration in the AD-DMT era

Implementing this model requires a shift towards synergistic collaboration between clinical disciplines. Optimal use of AD-DMTs requires integration of disease-specific expertise with a broader understanding of both cognitive change, and frailty and its implications.

In practice, this implies that frailty assessment should be considered a shared, cross-specialty competency involving clinicians engaged in dementia care—from primary care physicians to specialists in memory service, neurology, geriatrics, psychiatry, and other disciplines involved in the management of older patients.

Interdisciplinary models are well established in other areas of medicine. Collaborative care frameworks integrating specialty expertise –including geriatricians– have been successfully implemented in fields such as stroke, cardiology, orthopaedics, oncology, and emergency medicine [[108], [109], [110], [111]]. These approaches have been associated with improved outcomes, reduced complications, and more appropriate care for older adults with complex health conditions. Extending similar principles to dementia care, particularly in individuals assessed for or at risk of cognitive decline, represents a step forward in addressing the growing complexity of an ageing population.

### 4.4 A frailty-informed pathway for AD-DMT evaluation

**Fig. 3:**
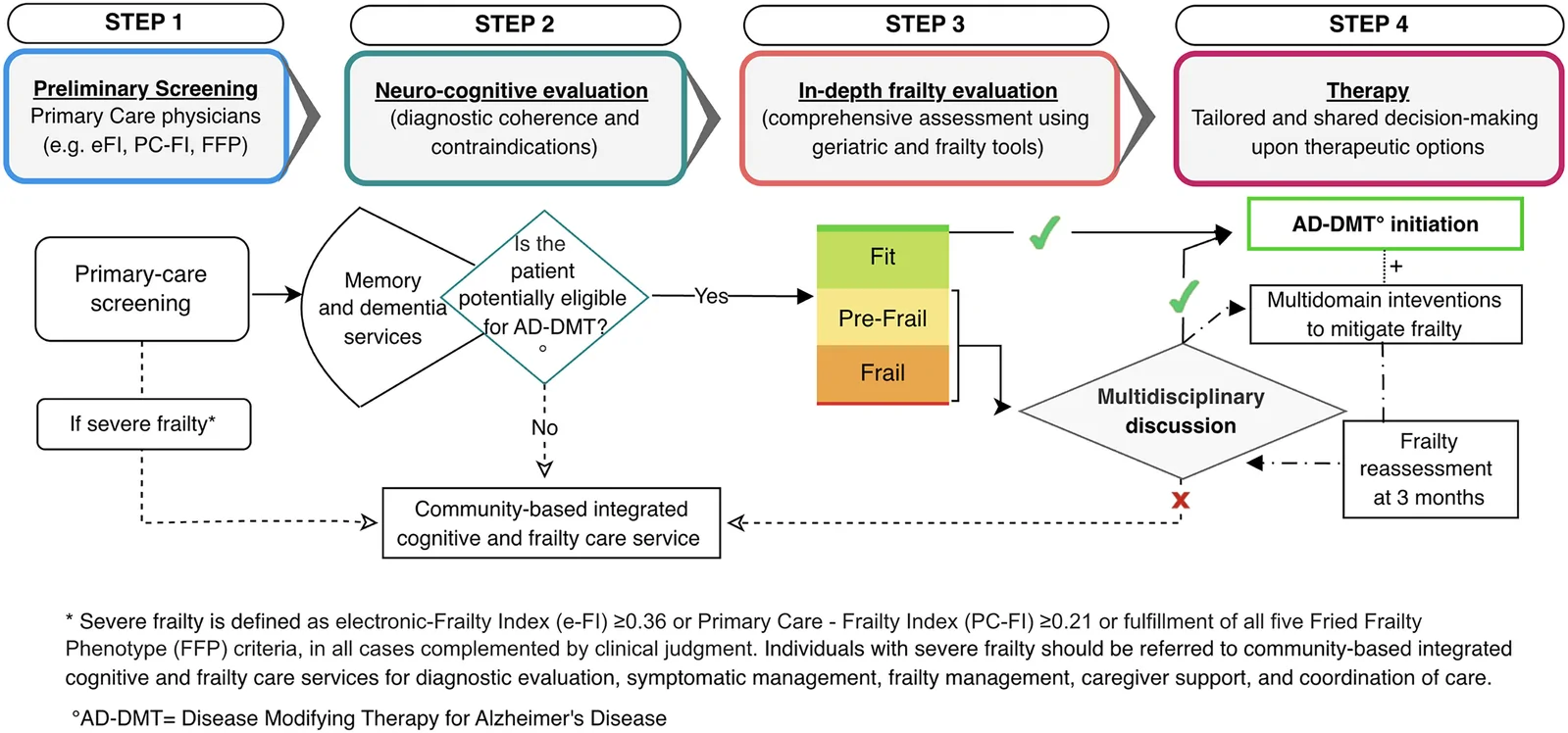
Frailty-guided framework to assess eligibility for Alzheimer’s disease-modifying therapies (AD-DMTs).

**Legend:** The proposed care pathway comprises three sequential steps. First, primary care physicians perform preliminary screening using appropriate tools and refer individuals to the appropriate care setting. Individuals with severe frailty should be referred to community-based integrated cognitive and frailty care services for diagnostic evaluations, cognitive and frailty management, caregivers’ support, and coordination of care. Second, memory and dementia services with expertise in AD-DMT administration perform a comprehensive neurocognitive and physical assessment, including evaluation of major contraindications, biomarkers, APOE genotype, and neuroimaging findings, to determine potential eligibility for treatment. Third, potentially eligible individuals undergo formal frailty assessment as part of a Comprehensive Geriatric Assessment, using appropriate tools to identify and grade frailty. Fit individuals may proceed directly to treatment, whereas those with pre-frailty or frailty undergo personalized multidisciplinary evaluation. Based on the overall benefit–risk profile, this may lead to treatment initiation, temporary deferral with targeted multidomain interventions, or AD-DMT initiation alongside frailty-mitigation strategies, followed by planned in-person or remote reassessment at appropriate intervals.

Fig. 3 illustrates a personalised, multidisciplinary diagnostic–therapeutic pathway for individuals potentially eligible for AD-DMTs, integrating frailty assessment within a staged evaluation process. A pragmatic approach to AD-DMT eligibility may begin with triage in primary care, where simple screening tools—such as e-FI—can help identify individuals with severe frailty, who are unlikely to benefit from extensive aetiological investigations and may be more appropriately managed through CGA and a holistic care approach. Severe frailty, in fact, carries a substantially increased risk of mortality from multiple causes [57,59] and reflects markedly reduced biological reserve, thereby diminishing the likelihood of meaningful benefit from AD-DMTs. For the purposes of the proposed framework, severe frailty may be operationally identified as e-FI ≥0.36, PC-FI ≥0.21, or fulfillment of all five FFP criteria, in all cases complemented by clinical judgment. These individuals should be referred to community-based integrated cognitive and frailty care services for diagnostic evaluations, cognitive and frailty management, caregivers’ support, and coordination of care. Individuals without severe frailty who may be potential candidates for AD-DMTs should instead proceed to memory and dementia services for specialist assessment of treatment eligibility. Given the high prevalence of mixed pathologies in older adults, particularly among PLwF, this assessment should carefully rule out the coexistence of non-AD pathology before determining AD-DMT eligibility [29,73,74]. Major contraindications should also be assessed, while formal frailty evaluation may help identify potentially modifiable domains and further inform treatment decisions. Individuals without clinically relevant frailty may then proceed to treatment, in accordance with approved clinical pathways and after comprehensive discussion of potential benefits and risks with the individual and their family. In the presence of pre-frailty and frailty, multidisciplinary discussion is essential to guide clinical decision-making. Potential strategies include proceeding with treatment, deferring therapy while implementing targeted multidomain interventions, or combining pharmacological treatment with interventions aimed at improving systemic resilience. The multidisciplinary assessment should also inform the timing of subsequent in-person or remote re-assessment, individualized according to frailty status, overall clinical condition, and changes occurring during treatment.

## 5 Future directions: research, trial design, and health system readiness

Future research should address the translational gap between evidence from randomized trials of AD-DMTs and the complexity of real-world clinical care, where frailty is highly prevalent and spans a broad spectrum of severity. Systematic integration of frailty assessment into research protocols and clinical pathways is therefore needed to establish its added value beyond standard clinical assessment ans usual care.

Prospective studies should incorporate validated frailty measures alongside clinical, functional, and biological variables to determine which instruments are most suitable for AD-DMT pathways and how they can best inform treatment selection and follow-up. Particular attention should be paid to distinguishing vulnerability captured by frailty measures from functional decline related to AD progression, especially in patients with mild dementia. Longitudinal studies should also establish whether frailty-informed care improves treatment effectiveness, tolerability, patient-centred outcomes, healthcare utilization, and cost-effectiveness.

Alongside inclusion of validated frailty measures in prospective studies, use of individual participant data meta-analysis (IPDMA) has potential to inform targeted treatment with AD-DMTs. IPDMA involves the collection, preparation, validation and analysis of the original participant-level trial data. It is a cornerstone of personalised medicine because it supports evaluation of the interaction between participant characteristics (e.g. frailty) and treatment effects (e.g. AD-DMTs) by increasing overall statistical power by pooling together data across trials, and has considerable potential in dementia and frailty research [112]. It has been demonstrated that it is possible to retrospectively operationalise frailty measures, typically using the FI, within randomised controlled trials of drug treatments across diverse disease areas, even if the original intention of the trial was not to identify frailty [113,114]. This approach has potential to support investigation of the relation between frailty, treatment effects, and potential for harm within AD-DMT trials. However, although a potentially attractive option, access to original trial data of AD-DMT trials and the necessary methodological expertise and resource required for this approach means that careful consideration and detailed planning would be required. In parallel to exploring access to historical trials of AD-DMTs, ensuring that future trials of AD-DMTs are designed to support future data sharing and harmonisation of data items to support future IPDMA would be a complementary step.

Progress in this field will require coordinated cross-disciplinary collaboration and the development of dedicated registries integrating frailty assessment within routine clinical data collection. Such infrastructures could provide real-world evidence on treatment safety, effectiveness, patient selection, healthcare utilization, and costs. Integration of frailty measures into electronic health records may also facilitate their implementation in routine care, although the feasibility and validity of automated approaches require further evaluation. Digital health technologies, including remote assessment, digital cognitive testing, and wearable-derived functional metrics, could improve accessibility and longitudinal assessmentwhile reducing patient and caregiver burden.

Future studies should also clarify the interplay between frailty, biological markers, and cognitive trajectories, to refine prognostic models and support more personalised treatment strategies.

Importantly, the clinical value of frailty-informed AD-DMTs care pathway cannot be established from cognitive and biomarker outcomes alone; patient-centred outcomes, including functional trajectories, resilience to health stressors, quality of life and treatment burden, should also be considered.

## 6 Conclusions

Frailty is a prevalent condition in the general population and contributes substantially to the biological and clinical complexity of AD dementia. It influences disease trajectories, treatment tolerance, and health outcomes, yet has rarely been systematically considered in the development or clinical evaluation of AD-DMTs.

At present, evidence is insufficient to determine whether PLwF should be systematically included or excluded from AD-DMTs. Frailty should not be interpreted as a simple contraindication. Instead, its systematic assessment may provide critical insight into potential treatment-related risks and may therefore serve as a clinical compass to guide therapeutic decisions and multidisciplinary discussion.

Optimal management will likely require the combining of pharmacological therapies with tailored multidomain interventions aimed at strengthening systemic and brain resilience. Improving physical function, optimizing comorbidity management, and addressing modifiable risk factors may enhance treatment tolerance and support patients’ capacity to cope with ongoing neurodegenerative processes. Integrating frailty assessment into research, clinical trials, and routine care pathways may help shift care from a disease-centred to a person-centred approach and will be essential to ensure that AD-DMTs and other treatments for AD are implemented safely, effectively, and equitably in this population.

## Funding source

none. The views expressed in this publication are those of the author(s) and do not reflect those of the funding institutions or any pharmaceutical companies.

## Declaration of generative AI and AI-assisted technologies in the manuscript preparation process

During the preparation of this work the authors used ChatGPT (Open-AI) in order to support language editing, manuscript structuring, and refinement of selected text passages. All AI-assisted content was critically reviewed and revised by the authors. The authors take full responsibility for the accuracy, integrity, and final content of the manuscript. Generative AI was not used as an author and did not independently determine the scientific content, positions, or conclusions presented in this paper.

## Data statement

No new data were generated or analyzed for this review.

## CRediT authorship contribution statement

**Giuseppe Bellelli:** Writing – review & editing, Writing – original draft, Conceptualization. **Ovidio Brignoli:** Writing – review & editing. **Marco Canevelli:** Writing – review & editing. **Antonio Cherubini:** Writing – review & editing. **Andrew Clegg:** Writing – review & editing. **Bruno Dubois:** Writing – review & editing. **Maria Cristina Ferrara:** Writing – review & editing, Writing – original draft. **Laura Fratiglioni:** Writing – review & editing. **Giovanni B. Frisoni:** Writing – review & editing. **Giulia Grande:** Writing – review & editing. **Frank Jessen:** Writing – review & editing. **Sean P Kennelly:** Writing – review & editing. **Alberto Magni:** Writing – review & editing. **Martina Marelli:** Writing – review & editing, Writing – original draft. **Alessandra Marengoni:** Writing – review & editing. **Nicolás Martínez Velilla:** Writing – review & editing, Writing – original draft. **Susan D. Shenkin:** Writing – review & editing. **Marc Suárez-Calvet:** Writing – review & editing. **Suzanne Timmons:** Writing – review & editing. **Alessandro Padovani:** Writing – review & editing, Conceptualization.

## Declaration of competing interest

The authors declare the following financial interests/personal relationships which may be considered as potential competing interests:

Giuseppe Bellelli reports a relationship with Eli Lilly that includes: consulting or advisory and speaking and lecture fees. Giuseppe Bellelli reports a relationship with IQVIA that includes: consulting or advisory. Marc Suarez Calvet reports a relationship with Roche Diagnostics that includes: consulting or advisory, funding grants, and speaking and lecture fees. Marc Suarez Calvet reports a relationship with Eli Lilly that includes: consulting or advisory and speaking and lecture fees. Marc Suarez Calvet reports a relationship with Grifols Inc that includes: consulting or advisory. Marc Suarez Calvet reports a relationship with Novo Nordisk that includes: consulting or advisory and speaking and lecture fees. Marc Suarez Calvet reports a relationship with Almirall that includes: speaking and lecture fees. Marc Suarez Calvet reports a relationship with Quanterix Corp that includes: speaking and lecture fees. Marc Suarez Calvet reports a relationship with Biogen that includes: speaking and lecture fees. Marc Suarez Calvet reports a relationship with Beckman Coulter Inc that includes: speaking and lecture fees. Marco Canevelli reports a relationship with Eli Lilly that includes: speaking and lecture fees. Antonio Cherubini reports a relationship with IQVIA that includes: consulting or advisory. Bruno Dubois reports a relationship with Alzheimer Research Foundation that includes: funding grants. Giovanni B. Frisoni reports a relationship with Association Suisse pour la Recherche sur la Maladie d’Alzheimer (APRA) that includes: funding grants. Giovanni B. Frisoni reports a relationship with Fondation Segré that includes: funding grants. Giovanni B. Frisoni reports a relationship with Ivan Pictet that includes: funding grants. Giovanni B. Frisoni reports a relationship with Race Against Dementia Foundation that includes: funding grants. Giovanni B. Frisoni reports a relationship with Fondation Child Care, Genève that includes: funding grants. Giovanni B. Frisoni reports a relationship with Edmond J Safra Foundation that includes: funding grants. Giovanni B. Frisoni reports a relationship with Fondation Minkoff that includes: funding grants. Giovanni B. Frisoni reports a relationship with Fondazione Agusta, Lugano that includes: funding grants. Giovanni B. Frisoni reports a relationship with McCAll Macbain Foundation, Canada that includes: funding grants. Giovanni B. Frisoni reports a relationship with Nicole et Renè Keller, Genève that includes: funding grants. Giovanni B. Frisoni reports a relationship with Fondation AETAS, Genève that includes: funding grants. Giovanni B. Frisoni reports a relationship with Roche Pharmaceutical that includes: consulting or advisory, funding grants, and speaking and lecture fees. Giovanni B. Frisoni reports a relationship with OM Pharma that includes: funding grants. Giovanni B. Frisoni reports a relationship with EISAI Pharmaceuricals that includes: consulting or advisory and funding grants. Giovanni B. Frisoni reports a relationship with Biogen that includes: consulting or advisory, funding grants, and speaking and lecture fees. Giovanni B. Frisoni reports a relationship with Novo Nordisk Inc that includes: funding grants and speaking and lecture fees. Giovanni B. Frisoni reports a relationship with H2020 that includes: funding grants. Giovanni B. Frisoni reports a relationship with Innovative Medicines Initiative (IMI) that includes: funding grants. Giovanni B. Frisoni reports a relationship with Swiss National Science Foundation that includes: funding grants. Giovanni B. Frisoni reports a relationship with IMI2 that includes: funding grants. Giovanni B. Frisoni reports a relationship with Velux Foundation that includes: funding grants. Giovanni B. Frisoni reports a relationship with Diadem that includes: consulting or advisory. Giovanni B. Frisoni reports a relationship with Lilly that includes: consulting or advisory. Giovanni B. Frisoni reports a relationship with Synaptica that includes: consulting or advisory. Giovanni B. Frisoni reports a relationship with GE Healthcare that includes: speaking and lecture fees. Giulia Grande reports a relationship with Svenska Lakersallskapet that includes: funding grants. Giulia Grande reports a relationship with JPND that includes: funding grants. Giulia Grande reports a relationship with Strategic Research Area in Neuroscience at Karolinska Institutet that includes: employment. Giulia Grande reports a relationship with Health Effect Institute Research Committee that includes: consulting or advisory. Frank Jessen reports a relationship with Roche that includes: consulting or advisory, funding grants, and speaking and lecture fees. Frank Jessen reports a relationship with AbbVie Inc that includes: consulting or advisory. Frank Jessen reports a relationship with Eli Lilly that includes: consulting or advisory and speaking and lecture fees. Frank Jessen reports a relationship with Eisai Inc that includes: consulting or advisory and travel reimbursement. Frank Jessen reports a relationship with Grifols Inc that includes: consulting or advisory. Frank Jessen reports a relationship with Priavoid that includes: consulting or advisory. Frank Jessen reports a relationship with Sanofi that includes: consulting or advisory. Frank Jessen reports a relationship with GE Healthcare that includes: speaking and lecture fees. Frank Jessen reports a relationship with Janssen-Cilag that includes: speaking and lecture fees. Frank Jessen reports a relationship with AC Immune that includes: board membership. Frank Jessen reports a relationship with EADC that includes: non-financial support. Alberto Magni reports a relationship with Angelini Holding SpA that includes: speaking and lecture fees. Alberto Magni reports a relationship with Viatris that includes: board membership and speaking and lecture fees. Alberto Magni reports a relationship with Alfasigma SpA that includes: board membership and speaking and lecture fees. If there are other authors, they declare that they have no known competing financial interests or personal relationships that could have appeared to influence the work reported in this paper.
